# Bidirectional crosstalk between microglia and serotonin signaling in neuroinflammation and CNS disorders

**DOI:** 10.3389/fimmu.2025.1646740

**Published:** 2025-08-26

**Authors:** Yangchen Zheng, Limin Xu

**Affiliations:** ^1^ School of Gongli Hospital Medical Technology, University of Shanghai for Science and Technology, Shanghai, China; ^2^ Department of Clinical Laboratory, Gongli Hospital of Shanghai Pudong New Area, Shanghai, China

**Keywords:** microglial activation states, serotonin (5-HT), serotonin transporter (SERT), neuroinflammation, neuropsychiatric disorders

## Abstract

Neuroinflammatory processes are increasingly recognized as central to the pathophysiology of diverse central nervous system (CNS) disorders, including major depressive disorder (MDD), Alzheimer’s disease (AD), and Parkinson’s disease (PD). Microglia, the resident immune effector cells of the CNS, are key regulators of neuroimmune responses and engage in bidirectional communication with the serotonergic system. Activation of microglia toward a pro-inflammatory phenotype can disrupt serotonergic neurotransmission by altering the expression and function of the serotonin transporter (SERT) and modulating downstream 5-HT receptor signaling pathways. Conversely, serotonergic neurotransmission—mediated through receptor subtypes such as 5-HT_1A_, 5-HT_2A_/_2B_, and 5-HT_7_—can regulate microglial phenotypic polarization and cytokine production, thereby influencing the inflammatory milieu and CNS homeostasis. This review synthesizes current evidence on the dynamic interplay between microglial activation states and serotonergic signaling, emphasizing their mutual contributions to disease onset and progression. Furthermore, we examine the therapeutic potential of targeting this neuroimmune interface using pharmacological strategies, including selective serotonin reuptake inhibitors (SSRIs), anti-inflammatory agents, and receptor-specific ligands. Clarifying this bidirectional crosstalk may inform the development of innovative interventions for neuroinflammation-associated neuropsychiatric and neurodegenerative disorders.

## 1 Introduction

Neuroinflammation is increasingly recognized as a central pathogenic mechanism underlying a broad spectrum of central nervous system (CNS) disorders, including major depressive disorder (MDD), Alzheimer’s disease (AD), and Parkinson’s disease (PD) (1–3). Among the primary cellular mediators of neuroinflammatory responses, microglia—the resident immune cells of the CNS—exert a dual role in preserving neural homeostasis and driving disease progression, depending on their activation state (4).The dynamic polarization of microglia between pro-inflammatory (M1-like) and anti-inflammatory (M2-like) phenotypes has been shown to critically influence neuronal survival, synaptic integrity, and behavioral outcomes (5). Recent studies have further revealed that microglial activation not only sustains neuroinflammatory cascades but also interacts with key neurotransmitter systems involved in emotion regulation and cognitive performance (6).

The serotonergic system, classically associated with mood regulation and higher-order cognitive functions, has emerged as a crucial modulator of neuroimmune interactions (7, 8). Serotonin (5-hydroxytryptamine, 5-HT) exerts immunoregulatory effects on microglia via subtype-specific receptor signaling pathways, while activated microglia release pro-inflammatory cytokines that can disrupt multiple aspects of serotonergic signaling—including alterations in serotonin transporter (SERT) activity as well as changes in 5-HT receptor expression, localization, and downstream signaling dynamics (9–11). This bidirectional interplay between serotonergic pathways and microglial function forms a regulatory axis that is essential for maintaining CNS homeostasis and is increasingly implicated in the pathogenesis and progression of both neuropsychiatric and neurodegenerative disorders (12, 13).

This review integrates current findings on the reciprocal regulation between microglial activation and serotonergic pathways, with a focus on how microglial phenotypic states influence both SERT and 5-HT receptor signaling, and how serotonergic receptors—such as 5-HT_1A_, 5-HT_2A/2B_, and 5-HT_7_—modulate microglial inflammatory responses. We further discuss the implications of this neuroimmune crosstalk in disease-relevant models and highlight emerging pharmacological strategies that target this interface as potential therapeutic approaches for MDD, AD, and PD.

## 2 Activation of Microglia and Neuroinflammation

Microglia, the primary immune effector cells of the CNS, play a vital role in initiating and regulating neuroinflammation (14). Their activation is typically triggered by various danger-associated molecular patterns (DAMPs) and pathogen-associated molecular patterns (PAMPs) (15). These molecular cues are recognized by pattern recognition receptors (PRRs) on the surface of microglia, activating downstream signaling cascades that lead to phenotypic changes (16). This biochemical alteration leads to the liberation of pro-inflammatory cytokines and mediators that facilitate the initiation and advancement of neuroinflammatory processes (17).

### 2.1 Role of pattern recognition receptors

Microglia possess a wide array of PRRs, such as Toll-like receptors (TLRs), NOD-like receptors (NLRs), and RIG-I-like receptors (RLRs), which enable them to detect specific molecular patterns and initiate innate immune responses (18).

TLRs, particularly TLR4, are among the most well-characterized PRRs in microglia. TLR4 can detect ligands like lipopolysaccharide (LPS) and high-mobility group box 1 (HMGB1) (19). Upon activation, TLR4 signals through both MyD88-dependent and -independent pathways, leading to the activation of Nuclear factor kappa B (NF-κB) and Mitogen-Activated Protein Kinase (MAPK) pathways. This results in the transcription and release of pro-inflammatory cytokines such as Tumor necrosis factor-alpha (TNF-α), interleukin-1 beta (IL-1β), and interleukin-6 (IL-6) (20). For instance, in Alzheimer’s disease (AD) models, amyloid-beta (Aβ) oligomers activate TLR4 on microglia, significantly increasing the production of IL-1β and TNF-α and exacerbating neuronal damage (21).

NLRs are intracellular PRRs that detect bacterial peptidoglycans and cellular stress signals (22). Among them, the NLRP3 inflammasome is particularly crucial. Its activation involves two distinct steps: first, a priming signal such as LPS or TNF-α induces the NF-κB -mediated expression of NOD-like receptor family pyrin domain containing 3 (NLRP3) and pro-IL-1β; second, an activating signal like ATP or Aβ prompts the assembly of the inflammasome complex, leading to caspase-1 activation and the maturation and release of IL-1β and IL-18 (23). Recent findings strongly associate NLRP3 hyperactivation with the development of several neuropsychiatric conditions (24).

### 2.2 Microglial functional states and phenotypic diversity

Microglial polarization plays a central role in mediating neuroinflammatory responses under both physiological and pathological conditions (25). Depending on the surrounding microenvironment, microglia can adopt different activation states that influence their function in either promoting or resolving inflammation (26). Traditionally, microglial phenotypes have been categorized into two opposing states: the pro-inflammatory M1 phenotype and the anti-inflammatory M2 phenotype. This classification, though useful in many experimental settings, has limitations when applied to the complexity of CNS disorders.

M1 microglia are activated by signals such as interferon-gamma (IFN-γ), LPS or TNF-α, and are distinguished by increased expression of pro-inflammatory cytokines (e.g., IL-1β, IL-6, TNF-α) and chemokines (e.g., CCL2, CXCL10) (26). These cells are essential for pathogen phagocytosis and clearance of cellular debris; however, their hyperactivation can induce neurotoxicity (27). M1 microglia contribute to neuronal apoptosis and synaptic degeneration by releasing pro-inflammatory mediators such as IL-1β, TNF-α, and IL-6, which can exert neurotoxic effects when persistently elevated (28). For instance, in ischemic stroke models, M1 microglia exacerbate injury via TNF-α secretion (29). Additionally, they produce reactive oxygen species (ROS) and nitric oxide (NO) through enzymes such as NADPH oxidase (NOX) and inducible nitric oxide synthase (iNOS), leading to oxidative stress and mitochondrial dysfunction in neurons (30).

Conversely, M2 microglia are induced by signals including interleukin-4 (IL-4), interleukin-10 (IL-10), or transforming growth factor-beta (TGF-β), characterized by high expression of anti-inflammatory cytokines (e.g., IL-10, TGF-β) and neurotrophic factors (e.g., brain-derived neurotrophic factor (BDNF), insulin-like growth factor 1 [IGF-1]) (31). M2 microglia are pivotal in tissue repair and resolution of neuroinflammation, mediating these effects through the secretion of anti-inflammatory mediators and promoting regenerative processes. For example, in spinal cord injury models, M2 microglia facilitate axonal regeneration via IGF-1 secretion (32). They also maintain CNS homeostasis by phagocytosing cellular debris and pathogens.

Although the M1/M2 classification has been instrumental in shaping our understanding of microglial responses, emerging research has made it increasingly clear that microglial activation is far more fluid, heterogeneous, and context-dependent than previously appreciated. The widely used “resting” and “activated” descriptors have been challenged, as microglia remain metabolically and functionally active even under homeostatic conditions. They continuously monitor their environment, engaging in synaptic remodeling, phagocytosis, and immune surveillance. Thus, microglia do not transition from a static “resting” state to an “activated” one upon injury or disease, but rather shift across a dynamic spectrum of states in response to region-, age-, sex-, and stimulus-specific cues (33).

Advances in single-cell RNA sequencing and multi-omics technologies have revealed diverse microglial populations that cannot be fully captured by binary categories. For instance, microglia rarely exhibit exclusive M1 or M2 marker expression *in vivo*, and often co-express both pro- and anti-inflammatory genes. Distinct microglial states such as disease-associated microglia (DAM), interferon-responsive microglia (IRM), lipid-droplet accumulating microglia (LDAM), and ARG1+ microglia have been identified under different pathological and developmental contexts (33). DAM, first described in Alzheimer’s disease models, are characterized by downregulation of homeostatic markers like P2RY12 and CX3CR1, and upregulation of Trem2, Apoe, Axl, and Spp1. Their emergence depends on TREM2 signaling and is associated with amyloid plaque interaction and phagocytic activity (34). However, DAM are not a universal signature of disease; their transcriptional features vary across species, brain regions, and disease models.

Nonetheless, the M1/M2 framework remains valuable for understanding how microglial polarization influences neuroinflammation. The polarization states of microglia are highly plastic and are modulated by the neuroimmune microenvironment. During early stages of Alzheimer’s disease, microglia predominantly exhibit an M2 phenotype, contributing to Aβ clearance; however, as pathology advances, a phenotypic shift toward M1 occurs, intensifying neuroinflammation and neuronal injury (35). Furthermore, epigenetic mechanisms, including DNA methylation and histone modifications, influence microglial phenotypic transitions, adding an additional regulatory layer to their functional states (36).

### 2.3 Key mediators of neuroinflammation

During neuroinflammatory processes, microglia secrete a diverse array of inflammatory mediators that are integral to maintaining CNS homeostasis and mediating responses to neural injury. These mediators predominantly encompass pro-inflammatory cytokines, anti-inflammatory cytokines, chemokines and ROS, collectively constituting a complex regulatory network that orchestrates neuroimmune responses. Sustained release of these mediators may result in chronic neuroinflammation, thereby contributing to the pathogenesis and progression of neuropsychiatric and neurodegenerative disorders (37).

Pro-inflammatory cytokines are proteins extensively secreted by activated microglia, chiefly including TNF-α, IL-1β, and IL-6. These cytokines are pivotal in initiating and amplifying neuroinflammatory cascades. TNF-α functions as a central inflammatory mediator, primarily activating the NF-κB signaling pathway to induce the expression of additional pro-inflammatory factors and augment microglial activation (38). Moreover, TNF-α interacts with its receptor TNFR1 to induce neuronal apoptosis, thereby exacerbating neuronal degeneration in neurodegenerative conditions (39). IL-1β is processed and released following NLRP3 inflammasome activation in response to pro-inflammatory stimuli, promoting microglial chemotaxis and enhancing phagocytic activity, while also contributing to neuronal injury under pathological conditions (40). In Alzheimer’s disease models, IL-1β expression is upregulated following Aβ deposition and exacerbates neuroinflammation by amplifying glial reactivity and cytokine release, thereby accelerating disease progression (41). IL-6 displays pleiotropic effects, mediating pro-inflammatory signaling in acute neuroinflammation, while potentially exerting neuroprotective or anti-inflammatory roles under certain chronic or regulatory conditions. Evidence suggests that IL-6 activates the Janus kinase–signal transducer and activator of transcription (JAK/STAT) pathway to support neuronal survival; however, its persistent overexpression may lead to excessive glial proliferation and heightened neuroinflammation (42).

In the modulation of neuroinflammatory responses, anti-inflammatory cytokines such as IL-4, IL-10, and TGF-β serve to attenuate excessive inflammatory activity, facilitate neural tissue repair, and promote microglial polarization toward the M2 phenotype. IL-4 is a key regulator of M2 microglial polarization, activating the Signal Transducer and Activator of Transcription 6 (STAT6) pathway to upregulate anti-inflammatory gene expression, including Arg-1 and CD206 (43). IL-4 exerts neuroprotective effects during post-injury recovery by reducing neuronal damage and enhancing synaptic plasticity. IL-10, predominantly produced by microglia and astrocytes, inhibits NF-κB and MAPK signaling pathways, thereby decreasing pro-inflammatory cytokine production and supporting tissue regeneration (44). In various neuroinflammatory models, IL-10 has demonstrated efficacy in reducing inflammation and improving neurological outcomes. TGF-β is a multifunctional cytokine that plays a vital role in regulating neuroinflammation and promoting tissue repair. It activates the Smad signaling pathway to suppress inflammatory cytokine release and facilitate blood-brain barrier repair (45). Additionally, TGF-β significantly influences microglial activation states, limiting excessive M1 polarization and promoting a neuroprotective M2 phenotype.

Chemokines constitute a class of small molecular cytokines capable of modulating the chemotactic migration of immune effector cells and directing the trafficking and targeted activation of microglia within neuroinflammatory milieus. The C-C chemokine subfamily (CCL) and the C-X-C chemokine subfamily (CXCL) represent the predominant chemokine subclasses within the CNS (46). CCL2, also designated as monocyte chemoattractant protein-1 (MCP-1), functions as a principal chemotactic mediator in the CNS, effectively recruiting monocytes and microglia to inflammatory loci. In ischemic stroke models, CCL2 expression is markedly upregulated, facilitating inflammatory cell infiltration and aggravating neural tissue injury (47). CX3CL1, primarily expressed by neurons, interacts with its receptor CX3CR1, predominantly localized on microglia. The CX3CL1-CX3CR1 signaling axis is integral to maintaining microglial homeostasis and modulating their activation states (48). Disruption of CX3CR1 signaling results in microglial hyperactivation, thereby amplifying neuroinflammatory responses.

Reactive oxygen species (ROS) serve as critical signaling mediators during microglial activation and are central to neuronal injury mechanisms. The generation of ROS predominantly depends on mitochondrial respiratory function and the enzymatic activity of NADPH oxidases. NOX2, the principal source of ROS in microglia, can be activated by inflammatory stimuli to produce superoxide anions (O_2_^-^) and hydrogen peroxide (H_2_O_2_) (49). Several studies have shown that NOX2 expression is elevated in both human postmortem tissue and animal models of depression, implicating it in microglia-mediated oxidative stress and neuroinflammation associated with depressive symptoms (50, 51). Mitochondria produce ROS during oxidative phosphorylation; when mitochondrial integrity is compromised, ROS accumulation becomes excessive, further impairing neuronal and glial cell functions. Moreover, ROS contribute to neurodegeneration through oxidative modifications of DNA and proteins, thereby facilitating the progression of neurodegenerative disorders (52).

## 3 Serotonin

Serotonin, also referred to as 5-hydroxytryptamine (5-HT), functions as a vital monoaminergic neurotransmitter within the central and peripheral nervous systems (53). Its biosynthesis initiates with the essential amino acid L-tryptophan, acquired through dietary intake. The biosynthetic pathway involves two key enzymatic reactions: first, tryptophan hydroxylase (TPH) catalyzes the hydroxylation of L-tryptophan to produce 5-hydroxy-L-tryptophan (5-HTP) (54). TPH exists in two isoforms: TPH1, which is predominantly expressed in peripheral tissues such as the gut, and TPH2, which is mainly localized in the central nervous system. Subsequently, aromatic L-amino acid decarboxylase (AADC) decarboxylates 5-HTP to generate serotonin (55). Notably, approximately 90% of the body’s total serotonin is synthesized in the gastrointestinal tract by enterochromaffin cells and stored in platelets. In this context, peripheral serotonin primarily regulates gut motility via the enteric nervous system and contributes to vascular tone and hemostasis through platelet-mediated release (56). By comparison, serotonin in the CNS is synthesized locally by serotonergic neurons located in the raphe nuclei of the brainstem, where it plays crucial roles in mood regulation, cognition, and neuroimmune signaling.

Serotonin cannot cross the blood-brain barrier (BBB) under normal physiological conditions due to its polar nature and lack of a specific transporter (57). Therefore, central serotonergic activity depends on the transport of L-tryptophan across the BBB via carrier-mediated active transport mechanisms (58). Within the brain, L-tryptophan is converted into serotonin through a two-step enzymatic process and subsequently stored in neuronal synaptic vesicles. Upon neuronal depolarization and activation, serotonin is released into the synaptic cleft, where it interacts with postsynaptic receptors to modulate neural signaling (59). Most released serotonin is reabsorbed into the presynaptic neuron via the serotonin transporter (SERT). Inside the neuron, monoamine oxidase (MAO), primarily isoforms MAO-A and MAO-B, catalyzes the oxidative deamination of serotonin, producing 5-hydroxyindoleacetic acid (5-HIAA). As a primary metabolite, 5-HIAA is transported out of the CNS via active transport mechanisms and excreted through renal pathways. Synthesized serotonin is primarily stored in synaptic vesicles, remaining readily available for release upon neuronal stimulation (60).

### 3.1 Serotonin receptor

Serotonin exerts its effects through at least 14 receptor subtypes, most of which belong to the G protein-coupled receptor (GPCR) family, except for 5-HT_3_, which functions as a ligand-gated ion channel (61). These receptors regulate a range of neurophysiological processes, including mood regulation, cognitive function, sleep-wake cycles, and appetite control, through subtype-specific signaling mechanisms (62). In adult microglial cells, several serotonin receptor subtypes are expressed, particularly 5-HT_2A_, 5-HT_2B_, 5-HT_5A_, and 5-HT_7_, though their expression and roles may vary depending on the cellular environment and disease state (63).

The 5-HT_2A_ receptor, a member of the class A GPCR family, is widely regarded as a key therapeutic target in neuropsychiatry (64). Among serotonin receptor subtypes, 5-HT_2A_ is broadly expressed throughout the CNS, particularly within monoaminergic nuclei such as the dorsal raphe nucleus, median raphe nucleus, and ventral tegmental area—regions critical for mood regulation (65). In addition to mood regulation, 5-HT_2A_ modulates cognitive function by influencing glutamatergic neurotransmission, including interactions with NMDA receptor pathways. It also engages in crosstalk with other receptor systems, such as 5-HT_1A_, GABA-A, adenosine A_1_, and orexin OX_2_ receptors (66). 5-HT_2A_ antagonists represent a major class of psychotropic agents and are widely used in treating psychiatric disorders, notably through atypical antipsychotics such as clozapine, olanzapine, and risperidone (67). In addition to their use in schizophrenia, 5-HT_2A_ antagonists have been investigated as adjuncts to selective serotonin reuptake inhibitors (SSRIs) and other antidepressant therapies to enhance therapeutic outcomes in certain depressive disorders (65).

The 5-HT_2B_ receptor is a G protein-coupled receptor primarily coupled to Gq/11 proteins, initiating diverse intracellular signaling cascades upon activation. Upon stimulation, Gq/11 proteins activate phospholipase Cβ (PLCβ), which hydrolyzes phosphatidylinositol 4,5-bisphosphate (PIP_2_) to produce diacylglycerol (DAG) and inositol trisphosphate (IP_3_). These second messengers elevate intracellular calcium levels and activate protein kinase C (PKC) (68). Although the functional role of 5-HT_2B_ receptors in the CNS remains partially understood, emerging evidence suggests that SSRIs such as fluoxetine may indirectly influence neuroglial signaling by modulating astrocytic 5-HT_2B_ receptors, potentially contributing to their neurotrophic effects (69). While some studies have suggested that microglial 5-HT_2B_ activation may contribute to pro-inflammatory responses under pathological conditions (70), astrocytic 5-HT_2B_ signaling has been shown to enhance antioxidant capacity and stimulate the release of neurotrophic factors, such as S100β and BDNF, supporting a potential neuroprotective role (71). The role of 5-HT_2B_ receptors in neuroinflammation demonstrates considerable complexity and exhibits variation across different cell types and pathological conditions, indicating that their contribution to glial immune responses is context-dependent.

The 5-HT_7_ receptor is abundantly expressed in brain regions including the thalamus, hypothalamus, hippocampus, and cerebral cortex, primarily localized to neuronal somata and dendrites. It regulates circadian rhythms, sleep-wake cycles, and affective behaviors (54). Upon activation, 5-HT_7_ receptors engage both classical and non-canonical signaling pathways. The classical Gα_s_-mediated pathway activates adenylate cyclase, increasing Cyclic adenosine monophosphate (cAMP) and activating Protein Kinase A (PKA), which subsequently phosphorylates effectors such as ERK1/2 and Akt (72). The non-canonical Gα_12_-dependent pathway activates Rho GTPases (Rho, Rac, and Cdc42), facilitating dendritic remodeling and synaptogenesis (73). These pathways may also enhance BDNF signaling via modulation of TrkB receptors, contributing to synaptic plasticity and neuroprotection (73). Increasing evidence supports an anti-inflammatory role for 5-HT_7_ receptors in the CNS. In bacterial meningitis, 5-HT_7_ stimulation limited microglial overactivation and immune cell infiltration, preserving neuronal and vascular integrity (74). Protective effects against neuronal apoptosis and blood-brain barrier disruption have also been observed in Alzheimer’s disease models. These actions are partly mediated through modulation of glial responses (75). 5-HT_7_ receptors present significant therapeutic potential for treating neuroinflammatory and neuropsychiatric disorders through their capacity to simultaneously resolve inflammation and provide neuroprotection.

### 3.2 Serotonin transporter

The serotonin transporter (SERT) is a key regulator of serotonergic neurotransmission, mediating the high-affinity reuptake of serotonin from the synaptic cleft back into presynaptic terminals. This reuptake process is essential for terminating serotonergic signaling and maintaining synaptic homeostasis (76).

Dysregulation of SERT has been extensively implicated in the pathophysiology of affective disorders, particularly MDD and anxiety. Among the most extensively studied genetic variants, the serotonin-transporter-linked polymorphic region (5-HTTLPR) within the promoter of the SLC6A4 gene—especially the short (S) allele—has been consistently associated with reduced transcriptional activity, lower SERT expression, and increased susceptibility to stress-related psychopathology (77). Importantly, the behavioral and clinical consequences of this polymorphism are modulated by environmental and biological factors, including sex differences, early-life adversity, and perinatal exposure to SSRIs. These findings reveal a complex gene–environment interaction that influences emotional regulation and determines susceptibility to mood disorders (78).

SERT expression is subject to multilayered regulation encompassing transcriptional control, epigenetic modifications, post-translational processes, and protein–protein interactions. The SLC6A4 gene is located on chromosome 17q11.2 and is regulated by multiple intracellular signaling pathways (79, 80). Proinflammatory cytokines such as IL-1β and TNF-α have been shown to enhance SERT activity via p38-mitogen-activated protein kinase (MAPK)-dependent phosphorylation. This cytokine-mediated modulation is both time-sensitive and cell-type specific, and contributes to inflammation-induced alterations in serotonergic neurotransmission (81, 82). Post-translational mechanisms further refine SERT function and trafficking. For example, glycogen synthase kinase-3β (GSK-3β) enhances SERT membrane trafficking and transport activity via phosphorylation-dependent mechanisms. Additionally, kappa-opioid receptor ligands have been shown to modulate SERT function through noncanonical signaling pathways, while cyclic GMP can enhance SERT activity through mechanisms that do not involve direct phosphorylation of the transporter (83–85). Glycosylation is also essential for proper SERT folding, stability, and trafficking to the plasma membrane; mutations at N-glycosylation sites can disrupt these processes, resulting in diminished transporter function and reduced surface expression (86). Finally, SERT interacts with several intracellular proteins that fine-tune its surface expression and activity. For example, syntaxin 1A, a component of the SNARE complex, binds to the C-terminal domain of SERT and inhibits its transport activity by regulating its insertion into the plasma membrane (87).

## 4 Crosstalk between microglial polarization and serotonin signaling

**Figure 1:**
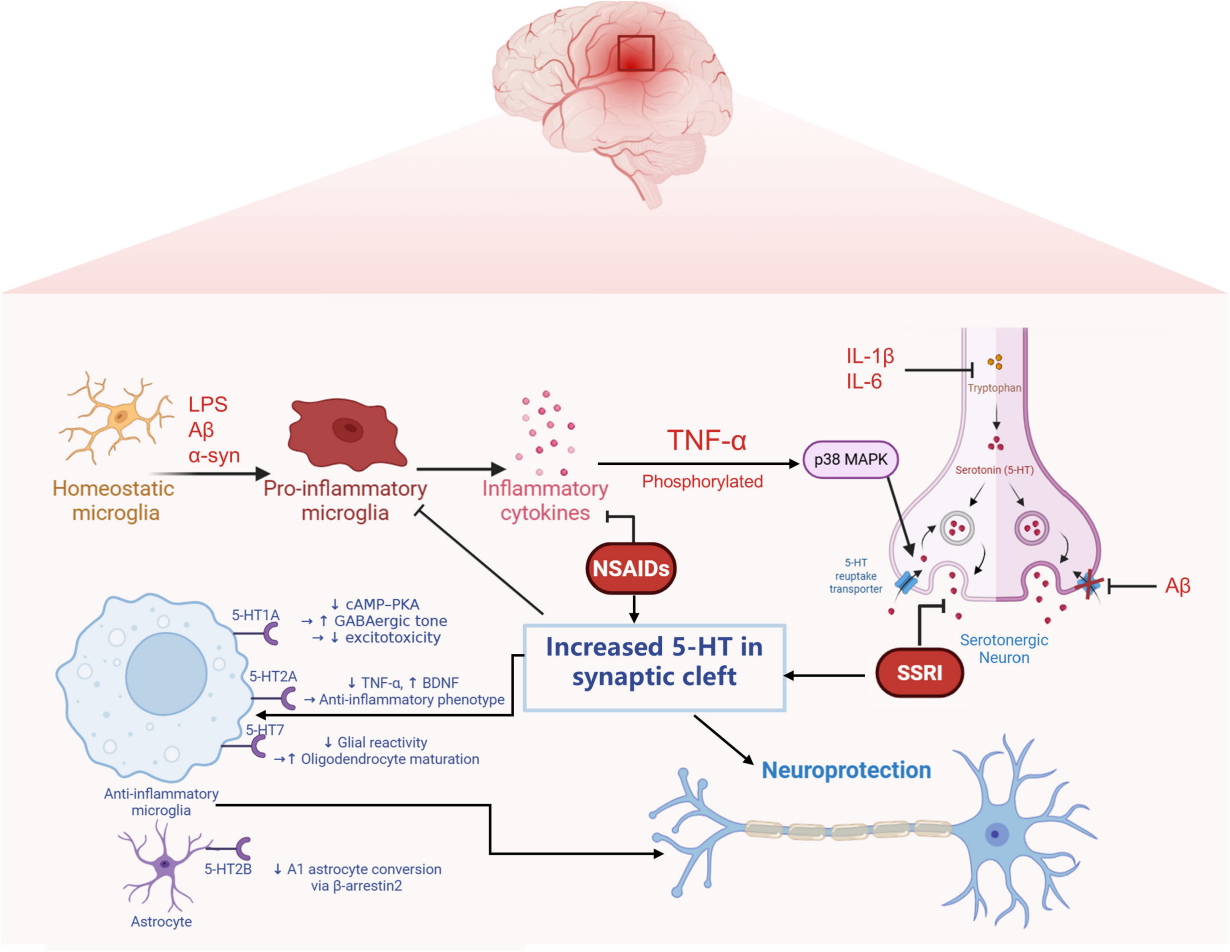
Reciprocal regulation between neuroinflammatory signaling and serotonergic transmission mediated by microglia and neurons. Pathological stimuli such as amyloid-β (Aβ) and lipopolysaccharide (LPS) activate microglia, leading to the release of pro-inflammatory cytokines including TNF-α, IL-1β, and IL-6. These cytokines stimulate intracellular signaling cascades such as p38 MAPK, which upregulates the expression of the serotonin transporter (SERT) in neurons, resulting in excessive serotonin (5-HT) reuptake and decreased extracellular 5-HT availability. Meanwhile, inflammatory signals also suppress the expression of tryptophan hydroxylase 2 (TPH2), the rate-limiting enzyme in 5-HT synthesis, further compromising serotonergic tone. Notably, SERT is subject to complex regulation under inflammatory conditions: while cytokines can enhance its expression via MAPK activation, Aβ has been shown to exert an opposing effect by downregulating SERT, indicating complex modulation of SERT.Conversely, 5-HT released into the synaptic cleft can act on specific receptors expressed on microglia to suppress their activation. Activation of the 5-HT1A receptor inhibits the cAMP–PKA signaling pathway, while 5-HT2B receptor engagement promotes β-arrestin2-dependent signaling. These receptor-mediated pathways collectively contribute to dampening microglial inflammatory responses. This bidirectional interaction reflects a dynamic feedback loop through which inflammation alters serotonergic homeostasis, while serotonergic signaling in turn modulates microglial activation states.

The interplay between microglial phenotypic polarization and serotonergic signaling is pivotal in the pathophysiology of neuroinflammation and neuropsychiatric conditions. Microglia-derived pro-inflammatory cytokines have been shown to influence SERT expression. In turn, the SERT/serotonin axis exerts a feedback effect on microglial inflammatory phenotypes through engagement of specific 5-HT receptors. This bidirectional neuroimmune communication is critically involved in the initiation and progression of both neuroinflammatory and neurodegenerative disorders (**Figure 1**).

### 4.1 Neuroinflammation regulation of the serotonin transporter

Activated microglia in the CNS release a spectrum of pro-inflammatory cytokines, notably TNF-α and IL-1β, which exert profound effects on the expression and function of the SERT. Recent studies suggest that these cytokine–SERT interactions are highly dynamic and regulated in a time- and cell type-dependent manner.

During the initial phase of the neuroinflammatory response, transient exposure to pro-inflammatory cytokines—particularly IL-1β and TNF-α—can rapidly modulate the function of the SERT through intracellular signaling cascades. Specifically, IL-1β binding to its receptor on serotonergic neurons activates the p38-MAPK pathway, resulting in increased transporter affinity for serotonin and enhanced reuptake efficiency within minutes (82). Similarly, TNF-α has been shown to transiently increase SERT surface expression and transport capacity via p38-MAPK signaling in various cellular models. These rapid molecular adaptations may lead to a temporary reduction in extracellular serotonin (5-HT) concentrations, contributing to behavioral manifestations such as lethargy and anhedonia (88, 89).

In contrast, models of chronic neuroinflammation—such as transgenic AD mice exhibiting sustained microglial activation and amyloid-β accumulation—demonstrate significantly reduced SERT activity in both cortical and hippocampal regions. This downregulation contributes to the serotonergic deficits commonly observed during disease progression (12). Conversely, rodent models of obesity-associated low-grade neuroinflammation display increased SERT expression in the hippocampus, accompanied by diminished serotonergic tone and attenuated therapeutic responses to selective serotonin reuptake inhibitors (SSRIs) such as escitalopram (90).

These findings demonstrate that SERT expression is upregulated during the initial phases of acute inflammatory responses and in conditions of chronic low-grade inflammation. Conversely, SERT expression becomes downregulated in environments marked by severe neuroinflammation. These observations raise the possibility that in some inflammation-associated depressive subtypes, such as post-stroke depression (91), the limited responsiveness to selective serotonin reuptake inhibitors (SSRIs) might, in part, reflect underlying neuroimmune dysregulation, warranting further investigation into this mechanistic link.

### 4.2 SERT/5-HT signaling feedback regulation of neuroinflammation

Serotonin receptor subtypes play a crucial role in regulating microglial activation within the CNS, a process that is fundamental to the development and progression of various neuroinflammatory and neuropsychiatric disorders. Notably, 5-HT receptors are expressed on both microglia and astrocytes, where they influence neuroimmune responses through distinct intracellular signaling pathways.

Recent research highlights the anti-inflammatory and neuroprotective roles of 5-HT_1A_ receptor activation across various central nervous system disorders. In a chronic glaucoma model, 8-OH-DPAT protects retinal ganglion cells by enhancing presynaptic GABAergic transmission through inhibition of the cAMP–PKA signaling cascade, thereby mitigating excitotoxicity and neuroinflammatory damage (92). Activation of the 5-HT_7_ receptor attenuates neuroinflammation in a neonatal mouse model of white matter injury by reducing glial reactivity and preserving oligodendrocyte maturation, suggesting its therapeutic potential in protecting the developing brain from preterm-related damage (93). Pharmacological activation of the 5-HT_2A_ receptor exerts immunomodulatory effects in the central nervous system by attenuating microglial TNF-α production and enhancing BDNF expression, thereby facilitating a shift toward an anti-inflammatory and neuroprotective microglial phenotype (94). The 5-HT_2B_ receptor plays a critical role in regulating neuroinflammation by modulating microglial developmental programming and immune reactivity, and its activation attenuates macrophage-driven neuroinflammatory responses in models of neuropathic pain and peripheral immune challenge (95, 96). Cytokines released by activated microglia—such as IL-1α, TNF-α, and C1q—can induce the formation of A1-type reactive astrocytes, which exhibit diminished neuroprotective properties and may contribute to neuronal damage (97). Activation of astrocytic 5-HT_2B_ receptors—for example, by fluoxetine—has been shown to block this phenotypic transformation via β-arrestin2-dependent mechanisms, thereby providing neuroprotection in experimental models of depression (71).

In summary, serotonergic signaling regulates glial activity in the CNS through receptor-specific mechanisms, shaping both neuroinflammatory responses and neuroprotective outcomes.

## 5 Microglial polarization and the interplay of 5-HT in disease pathogenesis

Pro-inflammatory polarization of microglia is a key driver of neuroinflammation and contributes to the pathogenesis of various neuropsychiatric disorders. Serotonin influences these processes by modulating microglial activation through specific receptor-dependent pathways. Recent evidence highlights a bidirectional relationship, whereby serotonergic signaling shapes microglial phenotypes, and inflammatory mediators from microglia in turn modulate components of the serotonergic system. This neuroimmune cross-talk represents a promising target for therapeutic strategies in psychiatric disease.

### 5.1 Depression

The etiology of depression is multifactorial, with the neuroinflammatory hypothesis gaining increasing prominence in recent years. Pro-inflammatory cytokines have been shown to induce core depressive symptoms, including anhedonia and reduced reward sensitivity (98). Experimental studies using rodent models demonstrate that intraperitoneal administration of LPS elevates central and peripheral cytokine levels, resulting in depression-like behaviors that can be attenuated by antidepressant treatment (99). Anti-inflammatory pharmacotherapies have also shown efficacy in reducing depressive symptoms, both in patients with comorbid inflammatory conditions and in those without (100). Non-steroidal anti-inflammatory drugs (NSAIDs), which inhibit COX enzymes and thereby suppress the synthesis of inflammatory mediators, have been reported to alleviate behavioral deficits induced by chronic unpredictable mild stress (CUMS) (101). Microglia polarized toward the pro-inflammatory M1 phenotype secrete large quantities of cytokines such as TNF-α, IL-1β, and IFN-γ, and their activation has been strongly linked to the pathophysiology of depression (102). Neuroimaging studies using positron emission tomography (PET), often combined with computed tomography (CT) or magnetic resonance imaging (MRI), have identified increased expression of the translocator protein (TSPO)—a marker of microglial activation—in the prefrontal cortex, anterior cingulate cortex, insula, and hippocampus of individuals with depression (103, 104). Notably, treatment with anti-inflammatory agents like minocycline has been shown to reverse these behavioral abnormalities and normalize microglial phenotypic imbalance, supporting the role of neuroinflammation in depression pathogenesis (105, 106).

The monoamine hypothesis remains a foundational framework for understanding depression, proposing that mood disturbances are driven by deficits in serotonergic, dopaminergic, and noradrenergic neurotransmission—particularly reduced serotonin in the prefrontal cortex, dopamine in the nucleus accumbens, and norepinephrine in limbic regions (107, 108). Consistent with this, neuroimaging studies have identified reduced activity and synaptic density in the dorsolateral prefrontal cortex (DLPFC) among patients with MDD (109, 110). Emerging evidence now points to a neuroimmune component, suggesting that dysfunction of astrocytic potassium channels and aquaporin-4 (AQP4) may disturb ion homeostasis, enhance neuronal excitability, and promote microglial polarization toward a pro-inflammatory M1 phenotype (111, 112). Inflammatory activation of glial cells leads to excess glutamate release, which may further disrupt prefrontal-limbic connectivity and dampen serotonergic transmission, providing a mechanistic link between neuroinflammation, glial dysfunction, and impaired 5-HT signaling in depression (113, 114). Moreover, diminished serotonergic activity may also be mediated by neuroinflammatory processes. Pro-inflammatory cytokines such as IL-1β and IL-6 can upregulate indoleamine 2,3-dioxygenase (IDO) activity, diverting tryptophan metabolism toward the kynurenine pathway. This results in the accumulation of neurotoxic metabolites, including quinolinic acid and kynurenic acid, which have been shown to impair neuronal function and reduce serotonin synthesis (115, 116). These findings suggest that M1-polarized microglia may contribute to serotonergic deficits in depression by both direct and indirect mechanisms.

Microglial activation is a central component of the neuroinflammatory processes implicated in depression and has been shown to modulate both the function and expression of the SERT. Pro-inflammatory cytokines released by activated microglia—particularly TNF-α, IL-1β, and IL-6—can regulate SERT via post-translational modifications and transcriptional mechanisms, thereby disrupting serotonergic neurotransmission within the CNS. Recent studies suggest that during acute inflammatory responses—such as transient exposure to TNF-α or IL-1β—SERT activity may be upregulated through p38 - MAPK -mediated phosphorylation, resulting in enhanced serotonin reuptake and a temporary reduction in synaptic 5-HT availability (89). This mechanism has been linked to behavioral phenotypes typical of early-stage depression, including anhedonia and motivational deficits. Animal models exposed to CUMS exhibit pronounced microglial activation in the prefrontal cortex and hippocampus, accompanied by region-specific changes in SERT expression (117). Some studies report SERT upregulation in the hippocampus under inflammatory conditions, while others describe reduced transporter availability in regions such as the amygdala (118).

Current pharmacological treatments for depression, including SSRIs and SNRIs, have been shown to modulate microglial activity and attenuate neuroinflammatory cascades. These agents suppress the production of pro-inflammatory mediators such as NO and ROS, thereby mitigating neuroinflammatory responses (119, 120). SSRIs primarily exert their antidepressant effects by inhibiting the SERT, thereby increasing synaptic serotonin concentrations (121–123). Elevated 5-HT levels enhance serotonergic neurotransmission and provide negative feedback on microglial cytokine release, including TNF-α and IL-1β (124, 125). In addition, SSRIs have been shown to promote neuroplasticity, in part through the upregulation of BDNF (126). Recent studies highlight the critical role of microglial polarization in mediating antidepressant responses. For instance, fluoxetine has been shown to inhibit the formation of neurotoxic A1 astrocytes by activating astrocytic 5-HT_2B_ receptors via a β-arrestin2-dependent pathway in mouse models of depression (71). Given that 5-HT_2B_ receptors are also expressed on microglia, it is plausible that SSRIs may exert direct anti-inflammatory effects on these cells. Similarly, fluvoxamine has been reported to induce a phenotypic shift in microglia from a pro-inflammatory M1 state to an anti-inflammatory M2 state in models of traumatic brain injury (127), although the precise mechanisms remain to be fully elucidated. Importantly, the interaction between the serotonergic system and neuroinflammation is bidirectional. While serotonergic pharmacotherapies can suppress microglial activation, inflammatory processes may simultaneously disrupt serotonergic homeostasis. Inflammation-induced activation of the kynurenine pathway depletes tryptophan—the precursor of serotonin—thereby reducing central 5-HT synthesis. Anti-inflammatory agents such as minocycline and NSAIDs have demonstrated the ability to restore serotonergic tone by elevating 5-HT levels and, in certain contexts, reducing aberrantly upregulated SERT expression under inflammatory conditions (128).

### 5.2 Alzheimer’s disease

Alzheimer’s disease (AD) is a progressive neurodegenerative disorder primarily characterized by age-related cognitive decline and memory impairment. Its hallmark neuropathological features include the accumulation of extracellular Aβ plaques and intracellular neurofibrillary tangles (NFTs) composed of hyperphosphorylated tau protein (129). Microglia, the resident immune cells of the CNS, respond to pathogenic stimuli such as aggregated Aβ peptides. Under physiological conditions, microglia exhibiting an M2-like phenotype are predominantly involved in the phagocytosis and clearance of insoluble fibrillar Aβ deposits, thereby exerting neuroprotective effects (130). However, increasing evidence suggests that chronic or dysregulated microglial activation leads to a shift toward a pro-inflammatory M1 phenotype, which contributes to neurotoxicity. M1-polarized microglia release pro-inflammatory cytokines and other neurotoxic mediators that can damage neurons directly or indirectly 想 by activating reactive astrocytes with neurotoxic A1-like properties (131). Notably, several Alzheimer’s disease (AD)-associated genetic risk variants—including APOE ϵ4, TREM2 R47H (rs75932628), CD33 rs3865444-C, INPP5D regulatory variants, MS4A6A locus variants, and PLCG2 P522R (rs72824905)—are enriched in microglia and influence their functional state (132). These variants have been linked to microglial processes such as activation, amyloid-β clearance, and inflammatory signaling. For example, APOE ϵ4 affects lipid handling and promotes a pro-inflammatory profile (133); TREM2 R47H reduces phagocytic capacity and impairs microglial survival (134, 135). CD33 and INPP5D variants are associated with altered inhibitory signaling thresholds, while the MS4A gene cluster modulates calcium dynamics and cytokine release (135). The rare PLCG2 P522R variant has been shown to enhance microglial immune reactivity and is thought to confer protection. These genetic factors are increasingly recognized as regulators of the transition from homeostatic to disease-associated microglial phenotypes in AD (136).

While cholinergic dysfunction has long been viewed as the primary neurochemical deficit underlying AD, growing evidence highlights a critical—and potentially upstream—role for serotonergic dysregulation in disease onset and progression (137, 138). Serotonin pathways regulate cognitive functions such as memory and learning by interacting with cholinergic, dopaminergic, and glutamatergic systems. Receptor subtypes including 5-HT_1A_, 5-HT_4_, 5-HT_6_, and 5-HT_7_ are abundantly expressed in cognition-related brain regions, with 5-HT_6_ receptors in particular being implicated in synaptic plasticity and cognitive enhancement (139–142). Moreover, 5-HT_2B_ receptor mRNA is increased in microglia within Aβ plaque-enriched regions of the cortex and hippocampus, although overall receptor binding appears reduced (143). Aβ aggregation, the hallmark pathological feature of AD, activates microglia and triggers the release of pro-inflammatory cytokines such as TNF-α and IL-1β. Both animal studies and human neuroimaging data showing reduced SERT availability in cortical regions of individuals with mild cognitive impairment (MCI) and AD (137, 144).

Pharmacologically, serotonergic agents—particularly SSRIs—have shown potential beyond mood regulation. Preclinical studies suggest that SSRIs can reduce amyloid burden and microglial activation, although clinical trials have yielded inconsistent results in established AD (145). Nonetheless, epidemiological data indicate that early SSRI use may delay the progression from MCI to AD (146). Vortioxetine, a multimodal antidepressant that targets both SERT and multiple 5-HT receptors, has demonstrated improvements in both mood and cognitive function in AD patients with comorbid depression (147).

### 5.3 Parkinson’s disease

Parkinson’s disease (PD) is the second prevalent neurodegenerative movement disorder among the elderly, characterized by tremors, motor deficits, and impairments in balance and coordination. Pathologically, it involves the degeneration of dopaminergic neurons within the substantia nigra pars compacta (SNpc) and the widespread intracellular aggregation of alpha-synuclein (α-syn) (148). The accumulation of α-synuclein can directly induce microglial polarization toward the pro-inflammatory M1 phenotype, thereby aggravating motor dysfunction and expanding neuronal injury in PD (149). Multiple preclinical investigations have demonstrated that peroxisome proliferator-activated receptor gamma (PPAR-γ) agonists facilitate microglial shift toward the anti-inflammatory M2 phenotype, potentially mitigating neuronal damage in PD (150). Although dopaminergic neuronal degeneration is central to PD pathology, impairments in serotonergic neurotransmission may also contribute to both motor and non-motor symptomatology (151, 152). Notably, 5-HT_1A_ receptor activation has been shown to alleviate levodopa-induced dyskinesia and modulate neuroinflammation, indicating a potential adjunctive role for serotonergic targets in PD therapy (153, 154). Reduced SERT availability in the raphe, assessed via SPECT imaging, has been associated with greater tremor severity and reduced responsiveness to dopaminergic therapy (155, 156). Therefore, serotonergic pharmacotherapies may offer therapeutic benefits for PD patients with resting tremors.

## 6 Existing drug repurposing

Repurposing clinically approved drugs for novel therapeutic indications represents a practical and cost-efficient approach. In the context of microglia–serotonin crosstalk, several anti-inflammatory and antidepressant compounds have demonstrated promising neuroimmune-modulatory effects in preclinical models. However, rigorous validation is required before their clinical applicability can be established.

Anti-inflammatory drugs, notably minocycline, have been tested in animal models of depression. In chronically stressed or LPS-challenged mice, minocycline prevented microglial activation, reduced neuroinflammation, and alleviated depressive-like behaviors, largely by inhibiting NF−κB signaling and promoting hippocampal neurogenesis (157). Additionally, minocycline showed selective inhibition of pro-inflammatory (M1) microglial polarization via NF−κB suppression *in vitro* and in models of neurodegeneration (158). Despite these neuroprotective effects, minocycline may cause side effects such as gastrointestinal upset, dizziness, pigment changes, and rarely, hypersensitivity or autoimmune reactions.

Antidepressant medications, especially SSRIs like fluoxetine, also modulate microglial activity. In LPS-stimulated BV2 microglial cultures, fluoxetine significantly inhibited production of TNF−α, IL−6 and NO by preventing NF−κB and p38 MAPK activation (159). Furthermore, in stroke and neurodegeneration models, fluoxetine boosted microglial phagocytosis and autophagy, reducing pro-inflammatory cytokine release. Notably, at higher doses or prolonged treatment, fluoxetine may increase oxidative stress in microglia—suggesting dose-dependent dual effects (160).

Most current evidence stems from rodent models or *in vitro* studies, which do not fully capture the complexity of human neuroimmune interactions. Differences in microglial phenotypes, serotonergic receptor distribution, and immune responses across species present challenges for clinical translation (161, 162). Long-term or off-label use of NSAIDs carries risks such as gastrointestinal bleeding, renal impairment, and cardiovascular events (163). Similarly, SSRIs may influence peripheral immune responses and potentially impair host defense, particularly with chronic or high-dose administration (164). Modulating microglial activity also raises concerns about unintended behavioral effects, including changes in cognition or stress adaptation (165). Inflammatory states may further interfere with SSRI efficacy, as elevated cytokine levels have been associated with poor antidepressant response. In light of these factors, while drug repurposing holds therapeutic promise, its clinical application requires careful evaluation (166). Future studies should incorporate inflammatory biomarkers, consider patient stratification, assess dose–response effects, and ensure long-term safety monitoring.

## 7 Conclusion

The interaction between microglial activation and serotonergic signaling represents a crucial regulatory axis in the development of neuroinflammatory and neuropsychiatric disorders. Pro-inflammatory cytokines released by activated microglia can modulate the expression and functional activity of the SERT through transcriptional, post-translational, and epigenetic mechanisms, thereby altering serotonergic tone within the central nervous system. In turn, serotonin receptors can influence microglial activation states, promoting either pro- or anti-inflammatory phenotypes and contributing to the regulation of neuroimmune homeostasis.

This bidirectional relationship holds significant therapeutic implications. Serotonergic agents, such as SSRIs, have been shown to dampen neuroinflammatory responses mediated by microglia, while certain anti-inflammatory compounds—including NSAIDs and tetracyclines—can restore SERT expression and improve serotonergic neurotransmission. These findings suggest a potential dual therapeutic strategy: enhancing serotonergic signaling to reduce inflammation and using anti-inflammatory approaches to support serotonergic function.

To further advance understanding of this interplay, future studies should focus on the spatial and temporal dynamics of microglial activation, serotonergic receptor expression, and SERT regulation across disease-relevant brain regions. Techniques such as single-cell RNA sequencing and *in vivo* imaging may help clarify how these processes evolve during disease progression and respond to pharmacological intervention. Targeting both microglial reactivity and serotonergic dysregulation in a coordinated manner may ultimately offer improved outcomes for disorders such as major depressive disorder, Alzheimer’s disease, and Parkinson’s disease.
